# Comparison of real-world efficacy and safety of nivolumab plus ipilimumab or pembrolizumab combined with platinum-based chemotherapy as first-line treatment for advanced non-small cell lung cancer in a German population

**DOI:** 10.1007/s00262-025-04244-4

**Published:** 2026-01-31

**Authors:** Moritz Weber, Alexander Freiherr von Hammerstein-Equord, Manuela Weinreich, Stefan Andreas, Achim Rittmeyer

**Affiliations:** 1 Abteilung für Pneumologie (Forschung und Lehre), Universitätsmedizin Göttingen, Göttingen, Germany; 2 Klinik für Herz-, Thorax- und Gefäßchirurgie (HTG), Universitätsmedizin Göttingen, Göttingen, Germany; 3 Lungenfachklinik Immenhausen, Immenhausen, Germany

**Keywords:** NSCLC, Nivolumab, Pembrolizumab, Ipilimumab, Germany, Real-world

## Abstract

**Introduction:**

In advanced non-small cell lung cancer (NSCLC), nivolumab plus ipilimumab with platinum-based chemotherapy (NIC) and pembrolizumab with platinum-based chemotherapy (PC) are commonly used treatment options. We conducted a single-center retrospective analysis comparing the two options under real-world conditions in Germany.

**Methods:**

We collected data from patients whose treatment was initiated between July 2018 and June 2023. Primary endpoints were overall survival and safety. Categorical data were compared using the chi-squared test or Fisher’s exact test. Overall survival was compared using the Kaplan–Meier method and the log-rank test.

**Results:**

200 patients, 77 treated with NIC and 123 treated with PC, were included. Baseline characteristics were unbalanced with significantly more older, squamous, and PD-L1-negative cases in the NIC group. Median overall survival (OS) and time to treatment discontinuation (TTD) were not significantly different in the NIC and PC groups (13.6 vs. 14.1 months / 5.8 vs. 6.2 months). Clinically significant adverse events (AEsi), related treatment discontinuations, and treatment-related deaths also did not differ significantly (*p* = 0.885/*p* = 1.000/*p* = 0.709). There were more immune-related AEsi in the NIC group (*p* = 0.001) and more chemo-related AEsi in the PC group (*p* < 0.001).

**Conclusions:**

NIC and PC seem to be equally supportable options in the treatment of advanced NSCLC. Further analyses are needed to validate our findings.

**Supplementary Information:**

The online version contains supplementary material available at 10.1007/s00262-025-04244-4.

## Introduction

Lung cancer remains the leading cause of cancer death worldwide [1]. The most common type is non-small cell lung cancer (NSCLC), which is frequently already too advanced for local therapy at the time of diagnosis [2–4]. If no targetable oncogenic alterations can be detected, the recommended first-line treatment for those with advanced NSCLC includes antibodies targeting various structures of immunomodulatory pathways like programmed cell death protein 1 (PD-1) or cytotoxic T-lymphocyte-associated antigen 4 (CTLA-4) [5]. In most cases, these immune checkpoint inhibitors (ICIs) are combined with platinum-based chemotherapy to further enhance the antitumor immune response, inducing mechanisms such as immunogenic cell death [6].

Two very common regimens for the first-line treatment of advanced NSCLC include the anti-PD-1-antibody pembrolizumab as well as the dual immune checkpoint inhibition with the anti-PD-1-antibody nivolumab and the anti-CTLA-4-antibody ipilimumab. These combinations have demonstrated their efficacy and safety in the pivotal trials KEYNOTE-189/-407 and CheckMate-9LA, respectively [7–9]. Choosing the optimal treatment, however, remains challenging as there are currently no official recommendations weighing the different options [10]. This is presumably because the current amount of real-world data and head-to-head studies comparing these two options regarding efficacy and safety is insufficient to draw a definitive conclusion.

Investigations previously conducted in Japan and Israel did not offer consistent answers, while in part raising awareness for a higher incidence of treatment-related deaths due to immune-related adverse events in actual practice [11–14]. Further investigations from other geographical regions are therefore warranted to complement the global body of evidence.

Here we present data from a single-center retrospective real-world study comparing efficacy and safety of patients with advanced NSCLC treated with nivolumab plus ipilimumab combined with two cycles of platinum-based chemotherapy (NIC) to pembrolizumab combined with four cycles of platinum-based chemotherapy (PC) at a specialized lung clinic in Germany.

## Materials and methods

### Study design and population

This was a single-center retrospective cohort study. It included patients with histologically confirmed NSCLC who received first-line treatment initiated between July 2018 and June 2023 with either nivolumab plus ipilimumab or pembrolizumab in combination with platinum-based chemotherapy based on the treatment regimens of CheckMate-9LA and KEYNOTE-189/-407, respectively, at Lungenfachklinik Immenhausen, a specialized lung clinic in Germany [7–9]. Allocation to treatment alternatives was based on the responsible physicians’ assessment of the patient’s clinical situation. Excluded were patients with activating EGFR mutations or ALK/ROS1 rearrangements. Cutoff date for data collection was August 31, 2024.

Data collected from medical records included information about age, sex, smoking status, stage of disease, previous antitumor treatment, histology, PD-L1 levels according to tumor proportion score (TPS) and immune cell score (IC), selected non driver mutations, choice of chemotherapy components, additional application of a concomitant radiotherapy (applied in parallel to and/or up to 6 months after last application of first-line treatment), outcome and safety.

### Assessment methods

Outcome was assessed by analyzing overall survival (OS), time to treatment discontinuation (TTD), and response rates. OS was defined as the time from treatment initiation until death from any cause. TTD was defined as the time from treatment initiation until the last application of first-line treatment before permanent discontinuation. Calculating OS and TTD in landmark analyses, the defined landmarks were used as starting point rather than the moment of treatment initiation. Treatment response was defined according to the Response Evaluation Criteria in Solid Tumors (RECIST), version 1.1 [15].

Safety was assessed by analyzing the incidence of adverse events, related treatment discontinuations, and treatment-related deaths (TRD). Adverse events of interest (AEsi) were those classified as clinically significant. Adverse events were considered clinically significant if they fulfilled at least one of the following criteria:

- Adverse events required the application of systemic immunosuppressive medication (e.g., prednisolone or infliximab)
- Adverse events required the alteration of therapy, meaning
  o Reduction of dose for at least one treatment component and/or
  o Temporary or partial discontinuation of treatment and/or
  o Permanent discontinuation of treatment
- Adverse events led to death

### Statistical analysis

Study groups were defined by choice of treatment: nivolumab plus ipilimumab with two cycles of platinum-based chemotherapy (NIC) or pembrolizumab with four cycles of platinum-based chemotherapy (PC). Comparisons between the two study groups were conducted using the chi-squared test for categorical data and, if the smallest expected value was < 5, Fisher’s exact test, respectively. Primary endpoints of this study were OS and safety. Secondary endpoints were TTD and the response rate, as well as OS and TTD in different subgroups. Survival was assessed using the Kaplan–Meier method with the log-rank test for comparison. The duration of follow-up was assessed using reversed Kaplan–meier estimations. Hazard ratios (HR) for OS and TTD were calculated using the Cox regression model. All statistical analyses were performed using IBM SPSS, versions 29.0 and 31.0, (Armonk, New Jersey, USA). The figures were created with the help of IBM SPSS, version 29.0 (Armonk, New Jersey, USA), Microsoft Excel (Redmond, Washington, USA) and Apple Pages (Cupertino, California, USA).

## Results

### Study population

200 patients were included, with 77 (38.5%) allocated to the NIC group and 123 (61.5%) to the PC group (Fig. S1). While there were no significant differences regarding sex, smoking history, stage of disease, metastatic locations, IC, and selected oncogenic mutations, significant differences in age distribution, histology, and TPS were observed. (Table 1).

**Table 1:** Baseline Characteristics compared between the treatment groups Abbreviations: NIC = nivolumab plus ipilimumab with two cycles of platinum-based chemotherapy, PC = pembrolizumab with four cycles of platinum-based chemotherapy, NSCLC = non-small cell lung cancer, PD-L1 = programmed death receptor ligand 1

	NIC group (n = 77)	PC group (n = 123)	*p*-value
**Age**, median [interquartile range]	70.8 [64.5-76.1]	68.2 [60.5-74.7]	
Older than 70 years, n (%)	42 (54.5)	47 (38.2)	*0.028*
**Sex**, n (%)			0.555
Male	44 (57.1)	76 (61.8)	
Female	33 (42.9)	47 (38.2)	
**Smoking history**, n (%)			0.216
Current or former smoker	64 (83.1)	110 (89.4)	
Never smoker	10 (13.0)	9 (7.3)	
unknown	3 (3.9)	4 (3.3)	
**Stage of disease**, n (%)			0.418
IV	60 (77.9)	102 (82.9)	
I - III	7 (9.1)	11 (9.0)	
Recurrent	10 (13.0)	9 (7.3)	
after operation	6	7	
after radiotherapy	4	2	
after systemic therapy	3	3	
unknown	0 (0.0)	1 (0.8)	
**Metastatic location**, n (%)			
Liver			0.204
yes	7 (9.1)	19 (15.5)	
no	70 (90.9)	103 (83.7)	
unknown	0 (0.0)	1 (0.8)	
Adrenal gland			1.000
yes	12 (15.6)	18 (14.6)	
no	65 (84.4)	104 (84.6)	
unknown	0 (0.0)	1 (0.8)	
Bone			0.157
yes	19 (24.7)	43 (35.0)	
no	58 (75.3)	79 (64.2)	
unknown	0 (0.0)	1 (0.8)	
Brain			0.474
yes	6 (7.8)	14 (11.4)	
no	71 (92.2)	108 (87.8)	
unknown	0 (0.0)	1 (0.8)	
**Histology**, n (%)			*< 0.001*
Adenocarcinoma	37 (48.1)	107 (87.0)	
Squamous cell carcinoma	35 (45.4)	15 (12.2)	
other NSCLC	5 (6.5)	1 (0.8)	
Large cell neuroendocrine carcinoma	2	1	
Adenosquamous carcinoma	2	0	
**PD-L1 level**, n (%)			
Tumor Proportion Score (TPS)			*0.019*
>= 50 %	1 (1.3)	12 (9.8)	
1-49 %	51 (66.2)	84 (68.3)	
< 1 %	25 (32.5)	25 (20.3)	
unknown	0 (0.0)	2 (1.6)	
Immune Cell Score (IC)			0.148
>= 10 %	6 (7.8)	13 (10.6)	
1-9 %	43 (55.8)	73 (59.3)	
< 1 %	25 (32.5)	23 (18.7)	
unknown	3 (3.9)	14 (11.4)	
**Selected oncogenic mutations**, n (%)			
Kirsten rat sarcoma virus (KRAS)			0.199
mutation	21 (27.3)	39 (31.7)	
wildtype	52 (67.5)	62 (50.4)	
unknown	4 (5.2)	22 (17.9)	
Tumor protein 53 (TP53)			0.150
mutation	38 (49.4)	37 (30.1)	
wildtype	32 (41.5)	52 (42.3)	
unknown	7 (9.1)	34 (27.6)	
Kelch-like ECH-associated protein 1 and/or Serin/Threonin-Kinase 11 (KEAP1 and/or STK11)			1.000
mutation	22 (28.6)	21 (17.1)	
wildtype	47 (61.0)	43 (34.9)	
unknown	8 (10.4)	59 (48.0)	

Significant differences were also noted regarding the application of chemotherapy components. Carboplatin was more commonly used as platinum component than cisplatin, with 68 (88.3%) patients in the NIC group and 69 (56.1%) in the PC group initially receiving carboplatin (*p* < 0.001). Pemetrexed was the more commonly used non-platinum component in the PC group, received by 104 (84.6%) patients, while in the NIC group (nab)paclitaxel was more frequently used, received by 56 (72.7%) patients (*p* < 0.001). Chemotherapy was completed without alteration by 56 (72.7%) patients in the NIC group and 57 (46.3%) in the PC group (*p* < 0.001).

A concomitant radiotherapy was initiated in 18 (23.4%) patients in the NIC group and 37 (30.1%) in the PC group (*p* = 0.332).

The median follow-up was 44.2 (32.8–55.7) months in the PC group and 22.1 (19.2–25.0) months in the NIC group (*p* < 0.001).

### Treatment efficacy

**Fig. 1:**
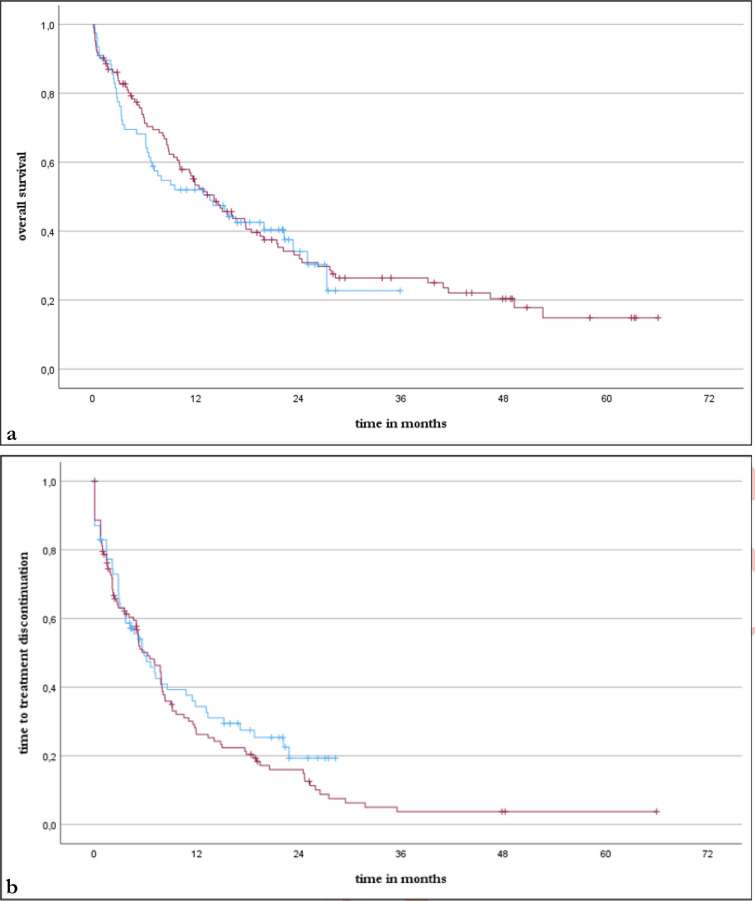
Kaplan–Meier curves of overall survival (a) and time to treatment discontinuation (b) for the NIC group (blue) and the PC group (red) Abbreviations: NIC = nivolumab plus ipilimumab with two cycles of platinum-based chemotherapy, PC = pembrolizumab with four cycles of platinum-based chemotherapy

Overall survival and time to treatment discontinuation did not differ significantly between the two treatment groups (Fig. 1). Median OS was 13.6 and 14.1 months (*p* = 0.719; HR: 1.069, CI95% 0.743–1.537) while median TTD was 5.8 and 6.2 months (*p* = 0.322; HR: 0.846, CI95% 0.605–1.184) in the NIC and PC groups, respectively. The response rates using the best overall response (BOR), 45.5% in the NIC group and 52.0% in the PC group, also did not demonstrate a significant difference (*p* = 0.386).

### Treatment efficacy in the subgroups

**Fig. 2:**
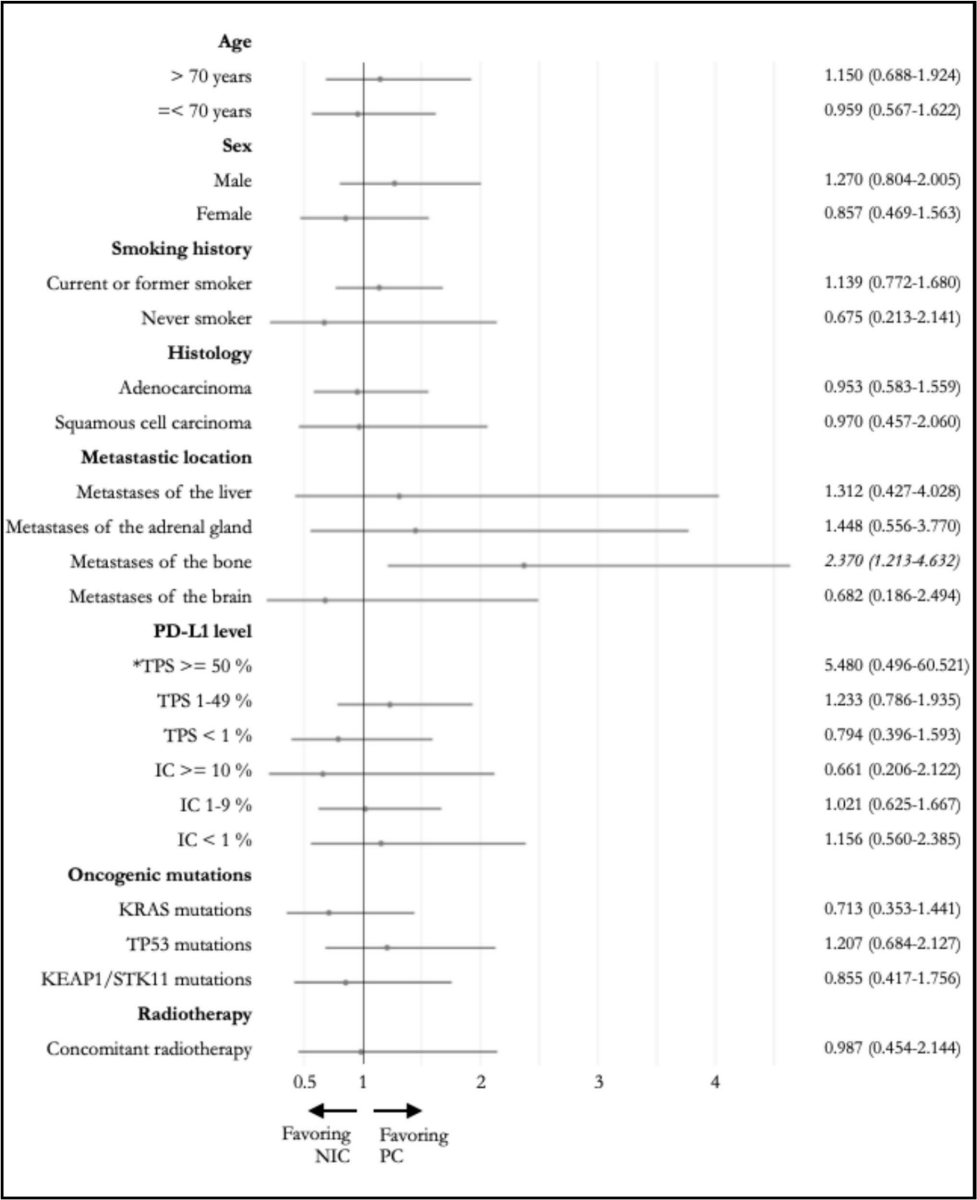
Forest plots and hazard ratios (with 95% confidence interval) for overall survival in different subgroups (*forest plot not depicted for reasons of clarity) Abbreviations: NIC = nivolumab plus ipilimumab with two cycles of platinum-based chemotherapy, PC = pembrolizumab with four cycles of platinum-based chemotherapy, PD-L1 = programmed death receptor ligand 1, TPS = tumor proportion score, IC = immune cell score, KRAS = kirsten rat sarcoma virus, TP53 = tumor protein 53, KEAP1 = kelch-like ECH-associated protein 1, STK11 = serin/threonine kinase 11

**Fig. 3:**
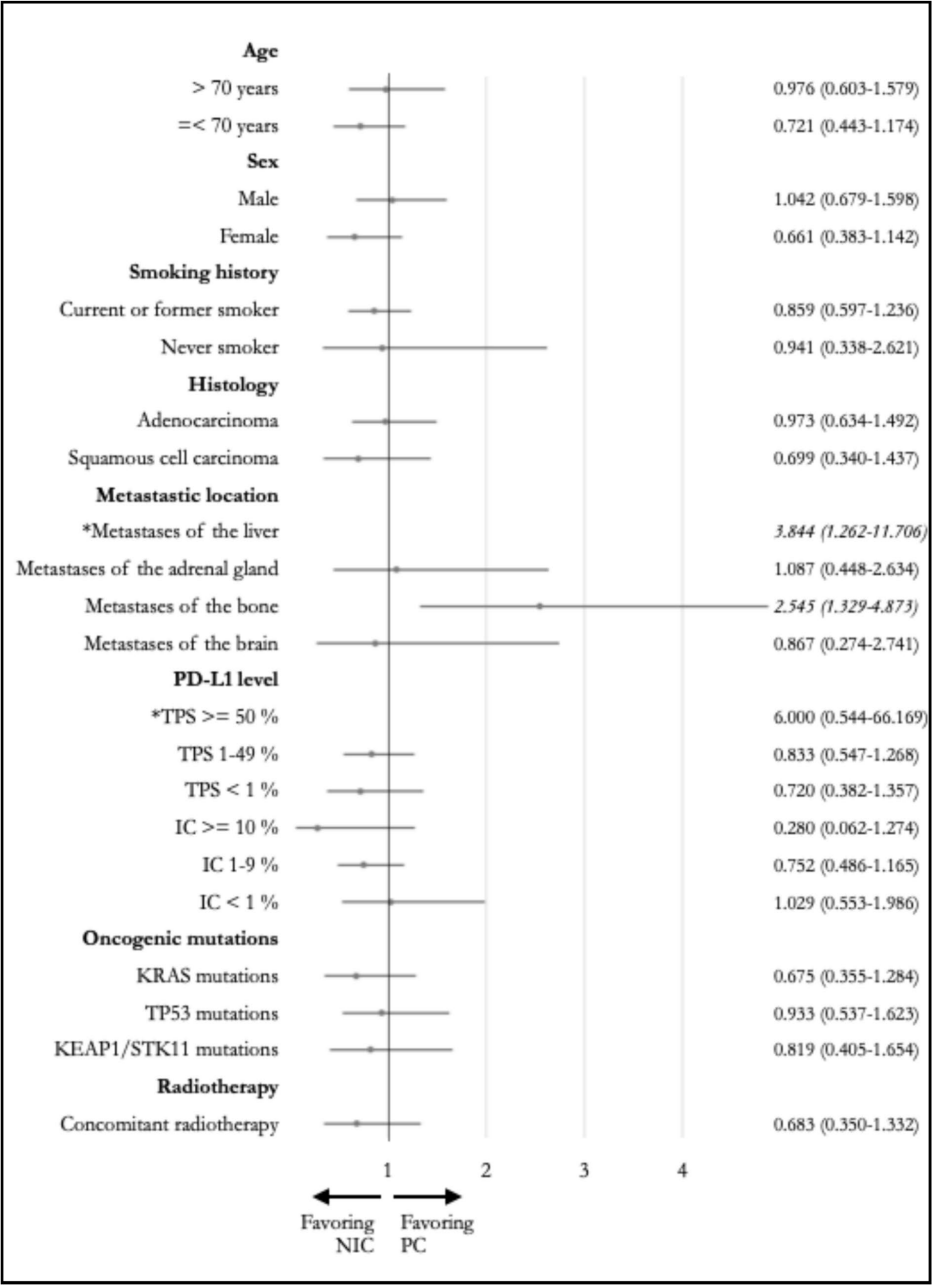
Forest plots and hazard ratios (with 95% confidence interval) for time to treatment discontinuation in different subgroups (*forest plot not depicted for reasons of clarity) Abbreviations: NIC = nivolumab plus ipilimumab with two cycles of platinum-based chemotherapy, PC = pembrolizumab with four cycles of platinum-based chemotherapy, PD-L1 = programmed death receptor ligand 1, TPS = tumor proportion score, IC = immune cell score, KRAS = kirsten rat sarcoma virus, TP53 = tumor protein 53, KEAP1 = kelch-like ECH-associated protein 1, STK11 = serin/threonine kinase 11

Among the analyzed subgroups, a significantly longer OS (Fig. 2) was observed in the PC group for those with bone metastases, with a median OS of 12.9 months compared to 3.7 months observed in the NIC group (*p* = 0.009; HR: 2.370, CI95% 1.213–4.632). TTD (Fig. 3) was also significantly longer in the PC group for this subgroup, with a median TTD of 6.2 months compared to 2.1 months observed in the NIC group (*p* = 0.003; HR: 2.545, CI95% 1.329–4.873). A significantly longer TTD in the PC group was also observed for those with liver metastases, with a median TTD of 5.1 months compared to 2.1 months observed in the NIC group (*p* = 0.009; HR: 3.844, CI95% 1.262–11.706). Median OS for this subgroup was not significantly different, with 3.7 and 8.1 months (*p* = 0.635; HR: 1.312, CI95% 0.427–4.028) in the NIC and PC groups, respectively.

No significant differences in OS (Fig. 2) and TTD (Fig. 3) were noted for the other subgroups of age, sex, smoking history, histology, metastatic location, PD-L1 level, and selected oncogenic mutations, as well as for those who received concomitant radiotherapy.

### Treatment safety

All observed AEsi are summarized in Table 2. AEsi occurred in 38 (49.4%) and 63 (51.2%) patients in the NIC and PC groups, respectively (*p* = 0.885). AEsi characterized as immune-related (irAE) occurred more frequently in the NIC group, where 32 (41.6%) patients developed irAEs compared to 25 (20.3%) in the PC group (*p* = 0.001). AEsi characterized as chemo-related (crAE) occurred more frequently in the PC group, where 46 (37.4%) patients developed crAEs compared to 11 (14.3%) in the NIC group (*p* < 0.001). The most common crAE and AEsi overall was hematotoxicity, which, like nausea/emesis and mucositis, occurred significantly more frequently in the PC group. The most common irAEs were immune-related skin reactions and pneumonitis, which occurred significantly more frequently in the NIC group (Table 2).

**Table 2:**
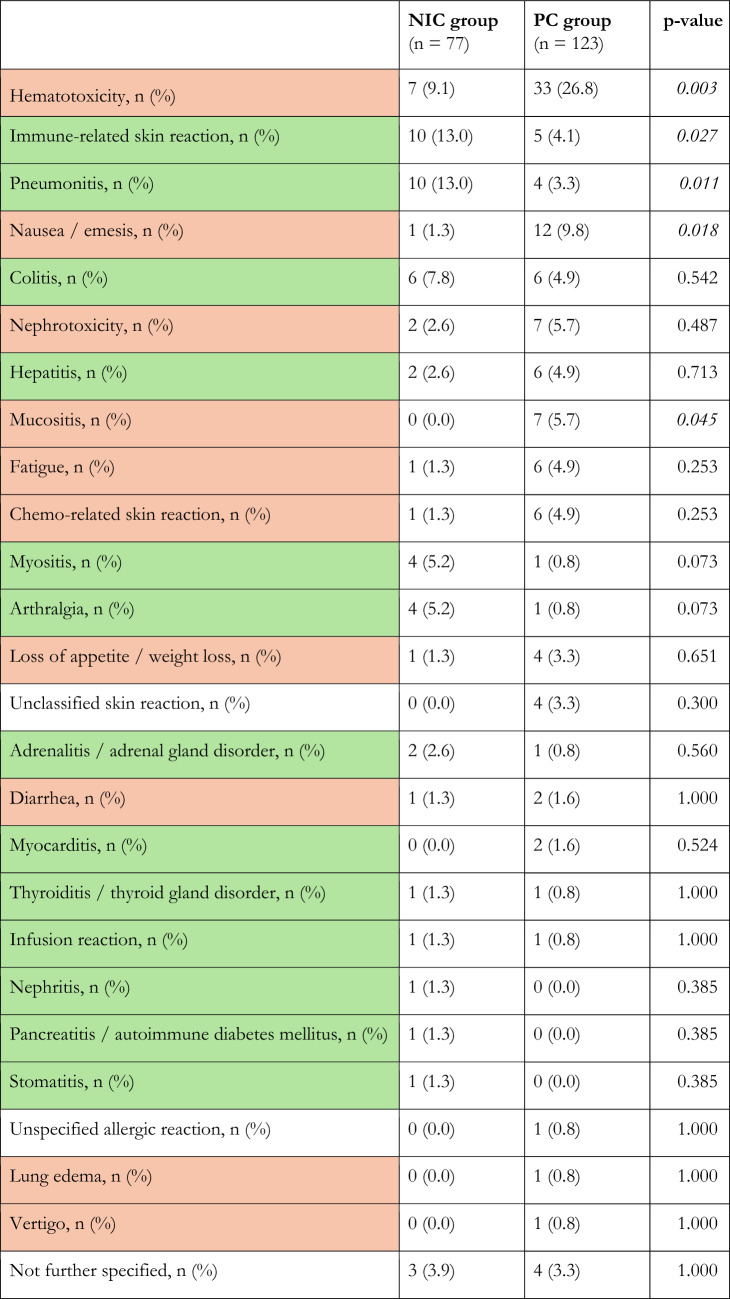
AEsi compared between the treatment groups with characterization as immune-related (green) or chemo-related (orange) if possible Abbreviations: NIC = nivolumab plus ipilimumab with two cycles of platinum-based chemotherapy, PC = pembrolizumab with four cycles of platinum-based chemotherapy, AEsi = adverse events of interest

13 (16.9%) patients in the NIC group and 21 (17.1%) in the PC group discontinued treatment permanently due to adverse events (*p* = 1.000).

There were 2 (2.6%) and 5 (4.1%) cases of TRD in the NIC and PC groups, respectively (*p* = 0.709) – all due to severe hematological AEs.

**Fig. 4:**
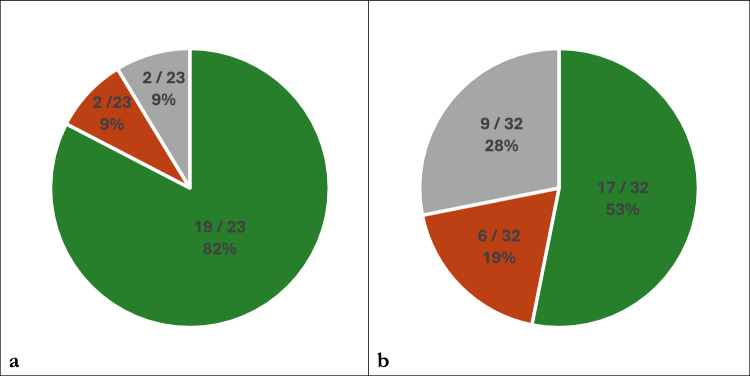
Classifications of cases with skin reactions according to attributed cause in the NIC group **(a)** and PC group **(b)** as immune-related (green), chemo-related (orange) or unspecified / both (grey) Abbreviations: NIC = nivolumab plus ipilimumab with two cycles of platinum-based chemotherapy, PC = pembrolizumab with four cycles of platinum-based chemotherapy

**Fig. 5:**
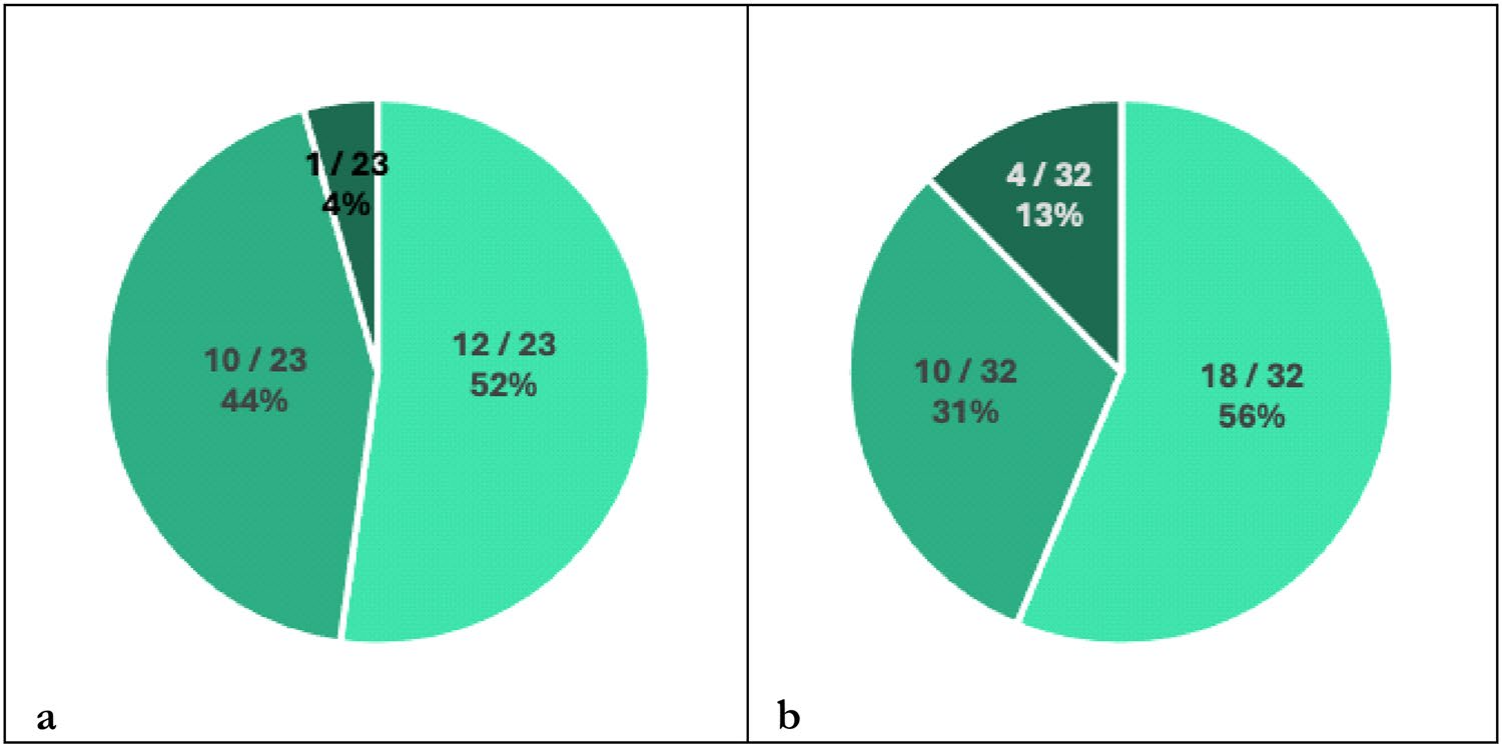
Classifications of cases with skin reactions according to severity in the NIC group **(a)** and PC group **(b)** as not clinically significant (light turquoise), clinically significant without permanent discontinuation of treatment (turquoise) or clinically significant with permanent discontinuation of treatment (dark turquoise) Abbreviations: NIC = nivolumab plus ipilimumab with two cycles of platinum-based chemotherapy, PC = pembrolizumab with four cycles of platinum-based chemotherapy

Skin reactions–regardless of cause and clinical significance–occurred in 23 (29.9%) patients in the NIC group and 32 (26.0%) in the PC group (*p* = 0.626). Classifications of skin reactions according to cause and clinical significance are shown in Figs. 4 and 5, respectively. The median time to onset overall was 1.0 month (interquartile range 0.7–3.5), with 2.8 months (interquartile range 0.7–4.9) and 0.7 months (interquartile range 0.7–2.9) in the NIC and PC groups, respectively.

### Association of skin reactions with treatment efficacy

**Fig. 6:**
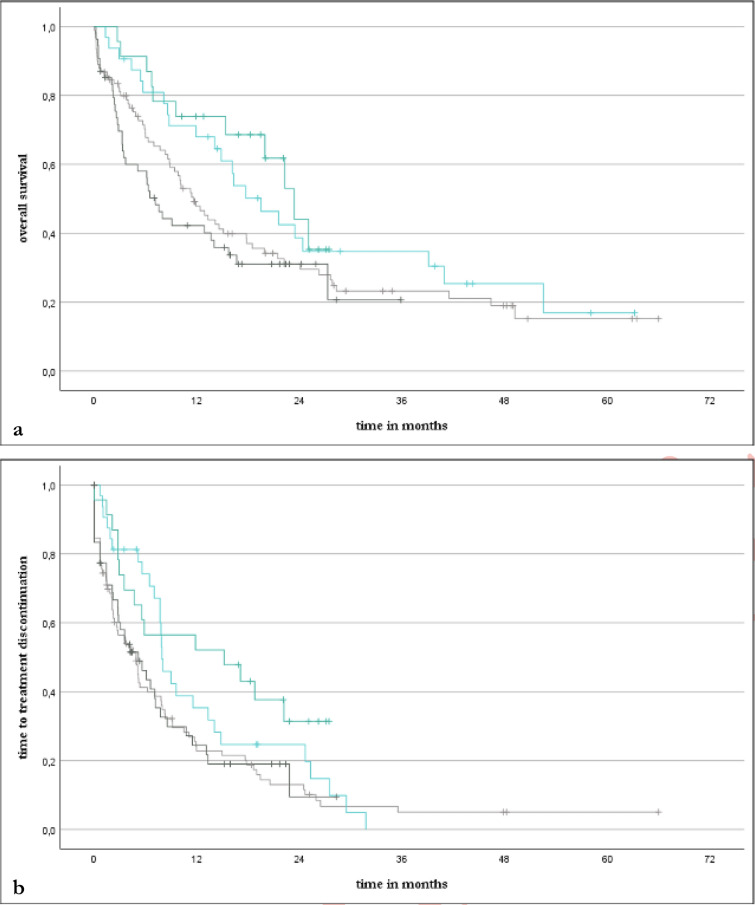
Kaplan–Meier curves of overall survival (**a**) and time to treatment discontinuation (**b**) for those in the NIC group with (dark turquoise) and without skin reactions (dark grey) as well as for those in the PC group with (light turquoise) and without skin reactions (light grey) Abbreviations: NIC = nivolumab plus ipilimumab with two cycles of platinum-based chemotherapy, PC = pembrolizumab with four cycles of platinum-based chemotherapy

OS in patients who developed skin reactions was significantly longer than in those who did not develop skin reactions (median OS 21.6 vs. 10.1 months, *p* = 0.014; HR: 0.612, CI95% 0.412–0.908). This trend was similarly observed for TTD (median TTD 9.0 vs. 4.9 months, *p* = 0.008; HR: 0.621, CI95% 0.434–0.887). Considering both treatment groups independently, median OS (23.4 vs. 7.1 months, *p* = 0.022; HR: 0.460, CI95% 0.233–0.908) and TTD (15.2 vs. 5.2 months, *p* = 0.035; HR: 0.526, CI95% 0.285–0.972) were also significantly longer for those with skin reactions in the NIC group alone. While median OS (19.5 vs. 11.6 months, *p* = 0.179; HR: 0.718, CI95% 0.441–1.167) and TTD (7.9 vs. 4.9 months, *p* = 0.141; HR: 0.721, CI95% 0.464–1.121) were also longer for those with skin reactions in the PC group, significance was not reached there (Fig. 6).

Landmark analyses, regarding the first onset of skin reactions, showed that patients who developed skin reactions demonstrated a median OS of 17.3 months and median TTD of 7.0 months. Considering both treatment groups separately, median OS and TTD were 19.3 months and 7.9 months in the NIC group and 16.7 months and 5.8 months in the PC group, respectively. Two cases in the PC group were not included in the landmark analyses of TTD as the first onset of skin reactions in these cases occurred after the last application of first-line treatment.

## Discussion

In our trial we observed no significant differences in short-term or long-term outcomes between the NIC and PC groups. This was despite significant differences of the study populations with a higher number of patients with older age, negative TPS and squamous histology in the NIC group. These characteristics have previously been associated with a trend towards worse outcomes in randomized controlled trials [7, 9, 16–18]. The follow-up period was significantly shorter for NIC due to lag in approval of the treatment alternatives. Stronger antitumoral effects of NIC compared to PC can therefore not be ruled out on the basis of this study.

In comparison, a real-world study conducted in Japan and an observational study from Israel recently demonstrated favorable OS for those treated with NIC compared to PC [11, 14]. However, another real-world analysis as well as a phase III trial (JCOG2007) from Japan and three previously published indirect analyses did—similar to us—not demonstrate significant differences in OS [12, 13, 19–21]. Our observed real-world outcomes, while similar regarding TTD, notably demonstrated shorter OS compared to the results of the pivotal trials leading to approval—especially for non-squamous NSCLC treated with PC [7–9, 16–18]. This discrepancy may be indicative of our real-world population having an already less favorable prognosis than the population enrolled in the clinical trials, presumably due to the application of less selective inclusion criteria and comparably older age, as well as a comparably shorter follow-up, which is similar to the assumptions of an ambispective Italian study comparing real-world data of PC in non-squamous NSCLC to the pivotal trial [22]. However, the aforementioned Japanese investigations also predominantly demonstrated longer OS compared to our findings [11–13]. This, on the other hand, may be explained by a general tendency towards more favorable outcomes among patients in East Asia and Japan compared to the overall population, as previously observed in subgroup analyses of KEYNOTE-407 and CheckMate-9LA [8, 23].

We also demonstrated no significant differences regarding the frequency of AEsi, related treatment discontinuations and TRDs between the treatment groups. Characteristics of AEsi, however, differed as significantly more irAEs were noted in the NIC group and significantly more crAEs in the PC group—highlighting the enhanced risk for cytotoxicity in the PC group due to the higher cumulative dose of chemotherapy as well as the enhanced risk for immunological reactions against healthy tissue in the NIC group due to the dual-use of ICIs targeting both PD-1 and CTLA-4. Hematotoxicity, was the cause of all TRDs in this study, whereby TRDs were not more common than in the approval trials [16, 24, 25]. This is a contrast to the findings of the Japanese studies, which demonstrated TRDs to be commonly caused by irAEs [11–13]. The analyses of Kaneko et al. and Shiraishi et al. at the same time observed TRDs to occur significantly more frequently in the NIC group, which in the JCOG2007 trial ultimately led to early termination of patient accrual [12, 13]. Findings of the East Asian and Japanese subgroup of the CheckMate-9LA population have likewise noted a higher incidence of irAEs compared to the overall population [23]. Therefore, it seems likely that safety profiles differ between geographical regions, putting those treated with NIC in East Asian ethnic groups at higher risk of developing irAEs with often more severe courses.

Another finding in our study was that OS and TTD for those with bone metastases were significantly longer in the PC group. This is in line with a previous retrospective study that suggested pembrolizumab might be more efficient in the treatment of bone metastases compared to other ICIs [26]. Further analyses of our data demonstrated that the number of patients receiving a concomitant radiotherapy addressing these metastases was higher – although not significantly –in the PC group (Table S1). It can therefore be speculated that the impact of the radiotherapy has influenced our results. Besides the local effects, abscopal effects could also play a role [27].

Skin reactions in both treatment groups were mostly not clinically significant, requiring only topical treatment or no medication at all. They occurred mostly within three months after treatment initiation and in only a few cases led to permanent discontinuation of treatment. On the other hand, skin reactions were associated with significantly longer TTD as well as OS. Additional landmark analyses also showed comparably longer survival times. These findings echo past retrospective observations [28, 29]. In NSCLC, T cells targeting shared antigens in cancer cells and skin cells may mediate tumor regression as well as autoimmune reactions against the skin—thereby suggesting them as potential biomarkers for response to ICIs [30]. In combination with chemotherapy, however, these immune-related skin reactions have to be differentiated from chemo-related forms caused by pemetrexed. This is highlighted by the fact that only in the NIC group, which demonstrated more immune-related and fewer chemo-related skin reactions compared to the PC group due to the aforementioned different toxicity profiles, the noted efficacy advantages remained significant.

This study has important limitations that need to be acknowledged. These include the retrospective design and the lack of randomization with the possibility of selection bias as well as the relatively small sample size. On the other hand, the unbalanced baseline characteristics reflect the choice of regimen under realworld conditions. Survival analyses demonstrate limitations due to the aforementioned different follow-up periods as well as in case of the TTD the structural differences of the study’s treatment regimes. In addition, the application of multiple comparisons in the subgroup analyses could have increased the risk for the occurrence of a statistical alpha error, masking false positive results [31]. Generalization of the results is also limited by the fact that the population was recruited from only a single center in Germany. Therefore, further studies including larger cohorts from different institutions in different geographical regions are warranted.

## Conclusion

Our study demonstrated similar efficacy for those treated with NIC and PC under real-world conditions. Safety profiles were also comparable regarding the incidence of AEsi and TRDs, while characteristics differed as NIC was more associated with irAEs and PC with crAEs. Overall, our findings suggest NIC and PC to be equally supportable options for the first-line treatment of advanced NSCLC without targetable oncogenic alterations. Further investigations are needed to validate our findings due to the small sample size and single-center design limiting generalizability.

## Supplementary Information

Below is the link to the electronic supplementary material.

Supplementary file1 (DOCX 71 KB)

## Data Availability

The presented data derive from data collected as part of the dissertation project (Doctor of Medicine) of Moritz Weber at the University Medical Center Göttingen. For this reason, the generated anonymized dataset will be stored for a period of ten years after submission of the thesis and can be made available upon well-founded request.
