# Correction: Comparison of real-world efficacy and safety of nivolumab plus ipilimumab or pembrolizumab combined with platinum-based chemotherapy as first-line treatment for advanced non-small cell lung cancer in a German population

**DOI:** 10.1007/s00262-026-04360-9

**Published:** 2026-04-06

**Authors:** Moritz Weber, Alexander Freiherr von Hammerstein-Equord, Manuela Weinreich, Stefan Andreas, Achim Rittmeyer

**Affiliations:** 1 Abteilung für Pneumologie (Forschung und Lehre), Universitätsmedizin Göttingen, Göttingen, Germany; 2 Klinik für Herz-, Thorax- und Gefäßchirurgie (HTG), Universitätsmedizin Göttingen, Göttingen, Germany; 3 Lungenfachklinik Immenhausen, Immenhausen, Germany

**Correction to: Cancer Immunology, Immunotherapy (2026) 75:65** 10.1007/s00262-025-04244-4

In the original version of this article, the acknowledgements section was incorrect and should have read

We would like to thank the Institute for Medical Statistics of the University Medical Center Göttingen and its Director Prof. Tim Friede for the financial support that has made this publication possible as well as for the introduction and consultation regarding the statistical methods used. We would also like to thank all participating patients and relatives, as well as the staff of the Lungenfachklinik Immenhausen We acknowledge further the support by the Open Access Publication Funds of the University of Göttingen.

