# Supplementary material for: Comparison of real-world efficacy and safety of nivolumab plus ipilimumab or pembrolizumab combined with platinum-based chemotherapy as first-line treatment for advanced non-small cell lung cancer in a German population
Source: Cancer Immunol Immunother. 2026 Jan 31;75(2):65. doi: 10.1007/s00262-025-04244-4 (PMC12860776; doi:10.1007/s00262-025-04244-4)
Supplement: Supplementary file 1 — Supplementary file1 (DOCX 71 KB) [file 262_2025_4244_MOESM1_ESM.docx]

Comparison of real-world efficacy and safety of nivolumab plus ipilimumab or pembrolizumab combined with platinum-based chemotherapy as first-line treatment for advanced non-small-cell lung cancer in a German population -

Supplementary Material

# Supplementary Figure 1: **Trial flow chart regarding selection of study population**


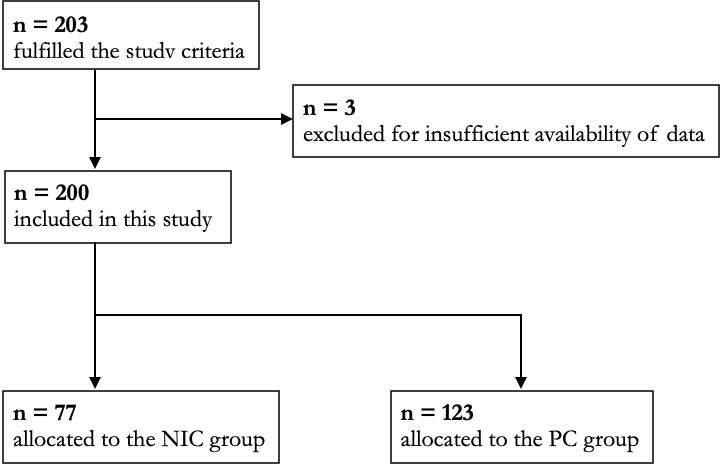


**Fig. S1** Trial flow chart regarding selection of study population This figure was created with the help of Apple Pages (Cupertino, California, USA). Abbreviations: NIC = nivolumab plus ipilimumab with two cycles of platinum-based chemotherapy, PC = pembrolizumab with four cycles of platinum-based chemotherapy

# Supplementary Table 1: **Concomitant radiotherapy addressing bone metastases in patients with bone metastases at baseline**

|  | **Patients with bone metastases at baseline**  (n = 52) | | |
| --- | --- | --- | --- |
|  | **NIC group**  (n = 19) | **PC group**  (n = 43) | **p-value** |
| Concomitant radiotherapy addressing bone metastases, n (%) | 4 (21.1) | 16 (37.2) | 0.252 |

**Table S1** Concomitant radiotherapy addressing bone metastases in patients with bone metastases at baseline Abbreviations: NIC = nivolumab plus ipilimumab with two cycles of platinum-based chemotherapy, PC = pembrolizumab with four cycles of platinum-based chemotherapy

# Supplementary Figure 2: **Relative frequency of times to onset of skin reactions in the NIC group (blue) and PC group (red)**

**Fig. S2** Relative frequency of times to onset of skin reactions in the NIC group (blue) and PC group (red) Abbreviations: NIC = nivolumab plus ipilimumab with two cycles of platinum-based chemotherapy, PC = pembrolizumab with four cycles of platinum-based chemotherapy, n.a. = not accessable
